# Translation of the Neck Disability Index and validation of the Greek version in a sample of neck pain patients

**DOI:** 10.1186/1471-2474-9-106

**Published:** 2008-07-22

**Authors:** Marianna N Trouli, Howard T Vernon, Kyriakos N Kakavelakis, Maria D Antonopoulou, Aristofanis N Paganas, Christos D Lionis

**Affiliations:** 1 Clinic of Social and Family Medicine, School of Medicine, University of Crete, Greece; 2 Department of Physiotherapy, University Hospital of Heraklion, Crete, Greece; 3 Canadian Memorial Chiropractic College, Toronto, Ontario, Canada; 4 Department of Orthopaedic Surgery and Traumatology, University of Crete, Greece

## Abstract

**Background:**

Neck pain is a highly prevalent condition resulting in major disability. Standard scales for measuring disability in patients with neck pain have a pivotal role in research and clinical settings. The Neck Disability Index (NDI) is a valid and reliable tool, designed to measure disability in activities of daily living due to neck pain. The purpose of our study was the translation and validation of the NDI in a Greek primary care population with neck complaints.

**Methods:**

The original version of the questionnaire was used. Based on international standards, the translation strategy comprised forward translations, reconciliation, backward translation and pre-testing steps. The validation procedure concerned the exploration of internal consistency (Cronbach alpha), test-retest reliability (Intraclass Correlation Coefficient, Bland and Altman method), construct validity (exploratory factor analysis) and responsiveness (Spearman correlation coefficient, Standard Error of Measurement and Minimal Detectable Change) of the questionnaire. Data quality was also assessed through completeness of data and floor/ceiling effects.

**Results:**

The translation procedure resulted in the Greek modified version of the NDI. The latter was culturally adapted through the pre-testing phase. The validation procedure raised a large amount of missing data due to low applicability, which were assessed with two methods. Floor or ceiling effects were not observed. Cronbach alpha was calculated as 0.85, which was interpreted as good internal consistency. Intraclass correlation coefficient was found to be 0.93 (95% CI 0.84–0.97), which was considered as very good test-retest reliability. Factor analysis yielded one factor with Eigenvalue 4.48 explaining 44.77% of variance. The Spearman correlation coefficient (0.3; P = 0.02) revealed some relation between the change score in the NDI and Global Rating of Change (GROC). The SEM and MDC were calculated as 0.64 and 1.78 respectively.

**Conclusion:**

The Greek version of the NDI measures disability in patients with neck pain in a reliable, valid and responsive manner. It is considered a useful tool for research and clinical settings in Greek Primary Health Care.

## Background

Neck pain is a highly prevalent condition among the general population. Data from cross-sectional studies show that point estimates range from 10% to 35% [1-3]. In a vast number of cases, there is no link between specific pathology and neck complaints, resulting in the term non-specific neck pain. The situation often leads to recurrences and chronicity, with a major impact on the quality of life of sufferers. In a recent prospective study assessing patients with non-specific back and neck pain seeking primary care, half of the respondents reported pain and disability at the 5-year follow-up [4]. In clinical trials, standardized scales are being used to capture important differences in disability, thus offering evidence for the effectiveness of one or another therapeutic intervention.

To our knowledge, five questionnaires measuring disability on a patient's life due to neck pain have been developed and validated [5]. The Neck Disability Index developed by Vernon and Mior [6] has been revalidated in several study populations and has shown stable psychometric properties [7-11]. In the past, a number of Greek authors have translated and validated questionnaires assessing musculoskeletal disorders [12,13]. However, no questionnaire assessing disability in activities of daily living in patients with neck pain has ever been validated in Greece.

The purpose of our study was the translation and validation of the NDI in a Greek sample with neck complaints seeking primary care. Our ultimate goal was to develop an instrument in Greek that would facilitate international research in musculoskeletal disorders as well as to serve health practitioners in their everyday clinical practice.

## Methods

### Questionnaire

The Neck Disability Index is a condition-specific instrument for self-report of disability. It is adapted from the Oswestry Low Back Pain Questionnaire [14]. It consists of 10 items referring to various activities (personal care, lifting, driving, work, sleeping, concentration, reading, recreation) and pain (pain intensity, headache) with 6 possible answers for each item. Patients are instructed to choose only one answer that most closely suits their condition at the present time. The score of each item varies between 0 (no pain and no functional limitation) and 5 (worst pain and maximal limitation) resulting in a total score of 0 (no disability) to 50 (totally disabled).

### Translation

The procedure was initiated after contacting the developer of the instrument and informing him about the purpose of the study. The translation strategy was selected based on minimal criteria developed by the Scientific Advisory Committee of the Medical Outcomes Trust [15]. Following these, two independent bilingual health professionals translated the questionnaire into Greek (forward translation). The mother tongue of both translators is the Greek language and their level of English is advanced. A reconciliation meeting was conducted to obtain a consensus version. Then, one native English speaker (an English teacher living and working in Greece for the last 15 years) who was blinded to the original version retranslated the re-conciliated Greek version into the source language (back translation). The back translation was sent to the developer and his suggestions were taken into account, thus formulating the revised Greek version of the Neck Disability Index (Gr -NDI). The duration of this phase was 1 month (10 March–10 April 2007).

**Figure 1:**
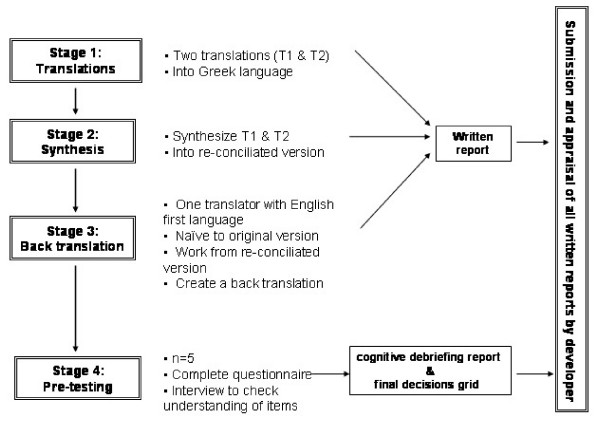
Graphic representation of the stages of the translation process.

The last step of the translation procedure was the pre-testing of the translated instrument in a small population of neck pain patients, using a cognitive debriefing process. This process refers to an in-depth interview of patients about their understanding of the questionnaire with the purpose of revealing inappropriate items and translation alternatives. Namely, after completing the questionnaire participants gave their general impression on the clarity of the items, the relevance of the content to their situation, the comprehensiveness of the instructions and their ability to complete it on their own. The same issues were addressed to them for every single item and they were able to make suggestions whenever necessary. Finally, a debriefing summary, including all participant interviews, and a final debriefing decisions grid were sent to the developer for comments. The duration of this phase was 1 month (10 April–10 May). Figure 1 demonstrates the flow of the translation process.

### Validation

#### Setting, sampling and target population

In order to explore the psychometric properties of the Greek version of the NDI, the questionnaire was administered to patients with neck pain, seeking primary care from 3 rural health centers. Patients eligible for the study were consecutively recruited from August to November 2007. Eligibility criteria were: age over 18, a written consent of the patient and absence of symptoms below the elbows related to specific neck disorders. Regarding the last criterion, patients with symptoms below the elbow and one positive finding in the conventional neurological testing and/or a positive Upper Limb Tension Test were excluded from the study.

#### Data collection

Eligible patients were informed for the purpose of the study and the confidentiality and anonymity of the process. After giving written consent they completed a questionnaire on demographic and clinical characteristics and the Gr-NDI. Patients visited the General Practitioners (GPs) one week later to complete the Gr-NDI with changed item order. According to Deyo [16], assessing reproducibility by retest at one-to-two week intervals (rather than a shorter interval), may result in more realistic estimates of the variability to be observed among control subjects in a longitudinal study. Patients also completed the Global Rating of Change (GROC), used as criterion for "stable" conditions.

The GROC rates the self-perception of change. Patients who state deterioration or improvement in a transitional scale, are asked to rate their condition from -7 (a very great deal worse) to -1 (almost the same, hardly any worse at all) and from 7 (a very great deal better) to 1 (almost the same, hardly any better at all) respectively [17].

#### Measurements

Data quality was assessed through completeness of data and floor/ceiling effects using the 15% criterion by McHorney [18]. Mean scores and standard deviations were calculated at item-level for both administrations of the NDI.

*Reliability *was assessed through internal consistency and test-retest reliability, as follows.

*Internal consistency *evaluates the extent to which items comprising a scale measure the same construct and was calculated using Cronbach's alpha. Values over 0.7 would be considered as acceptable [19].

*Test-retest reliability (reproducibility) *is the ability of an instrument to produce similar results on repeated administration when no real change in health status has occurred within this time frame [20]. Patients who scored between -3 and +3 on the GROC were included in the test-retest analysis, assuming that these patients had no clinically relevant changes [17]. Test-retest reliability was calculated using Intraclass Correlation Coefficient and Bland and Altman method [21]. The ICC is accepted as more appropriate than Pearson for quantifying reproducibility [22]. The size of the retest sample was estimated based on a method developed to calculate the required number of subjects in a reliability study [23]. Parameters regarding the probability of error type I and type II were α = 0.05 and β = 0.20 respectively. An ICC = 0.8 was defined as the minimal acceptable level of reliability and we hypothesized that our findings would be consistent with a minimum coefficient of 0.9. According to Nunnally [24] this level of reliability is the least still appropriate for person-level comparisons. Following these assumptions, 46 stable subjects were necessary for the test-retest analysis.

*Construct Validity *is the ability of an instrument to reflect a construct and was assessed through Exploratory Factor Analysis using a Varimax rotation [25].

*Responsiveness (sensitivity to change) *is the ability of a measuring instrument to detect clinically relevant changes over time [26]. It was analyzed by correlating the change score of the questionnaire to the GROC using the Spearman correlation coefficient. Deteriorated patients were excluded from the analysis (n = 2). Responsiveness was also assessed by the Minimal Detectable Change. The MDC expresses the minimal magnitude of change required to be 95% confident that the observed change between the two measures reflects real change and not just measurement error. It is calculated as 1.96 × v2 × SEM. Standard Error of Measurement is calculated as the square root of the within-subject variance of "stable" subjects [27]. Variance was computed with ANOVA for random effects. For all statistical analyses we used SPSS 15 for Windows.

#### Ethics

The study was approved by the Scientific Committee of the University Hospital of Heraklion (Protocol # 7213/1-8-2007).

## Results

### Translation

The developer comments on the translated tool concerned replacement of "pain" with "neck pain". This was applicable for items pain intensity, personal care and lifting. Two other comments about linguistic problems were back translation issues. The translated instrument was pre-tested on four women and one man with neck complaints. Their age ranged from 30 to 76 years and their educational level varied from elementary school to university. The general impression of the participants was that the questionnaire and the instructions were easy to understand and that the items were important to their situation. An older woman with low educational level stated that it was a bit difficult to complete and asked for explanations. The debriefing process also revealed difficulties of a single patient regarding 'lifting' and 'sleeping' items. She stated that her low back pain prevents her from lifting weights and that she does not sleep because of menopausal disturbances. Suggestions were not made since no modification could overcome such problems. Finally, two participants did not drive, resulting in missing data from this item. Since lifting, sleeping and driving are frequently susceptible to neck pain those items were not characterized as inappropriate. See additional file 1.

### Validation

Sixty-eight patients with neck complaints visited the Health Centers. Three patients did not meet the eligibility criteria and were excluded from the study. All eligible subjects agreed to participate in the study and returned to complete the questionnaires for a second time (100% response rate). Older patients asked for feedback from the GPs, which was consistent with the pre-testing findings. Descriptive statistics for missing patterns revealed six cases with at least two missing items, which were removed from all analyses. In addition, the score for cases with one missing item was adjusted by replacing the missing value with the median of the answers on the rest of the questionnaire. Demographic and clinical characteristics of patients and item-level descriptive statistics are presented in Tables 1 and 2 respectively.

**Table 1:** Demographic and clinical characteristics of the studied population

	**Mean**	**SD**
**Age **(years)	62,3	14,6
	**Frequency**	**%**
**Sex/Female**	36	55.4
**Male**	29	44.6
**Education/Illiterate**	6	9.4
**Elementary**	36	56.3
**High school**	8	12.5
**Lyceum**	7	10.9
**Higher ed**.	5	7.8
**University**	2	3.1
**Duration of last episode/Acute (1–7 days)**	42	65.6
**Sub-acute (7 days-7 weeks)**	11	17.2
**Chronic (>7 weeks)**	11	17.2
**Previous episodes/None**	5	12.5
**1–10**	14	35.0
**>10**	21	52.5
**Trauma/Yes**	4	6.3
**No**	60	93.8
**Neck surgery/Yes**	1	1.5
**No**	64	98.5
**Co-morbidity/Yes**	53	82.8
**No**	11	17.2

**Table 2:** Item descriptive statistics.

	***Day 1***			***Day 8***		
**Item**	**Missing (%)**	**Mean**	**SD**	**Missing (%)**	**Mean**	**SD**

**Pain intensity**	0.0	0.95	1.07	0.0	0.63	0.96
**Personal care**	0.0	0.72	0.94	0.0	0.55	0.88
**Lifting**	10.8	1.81	1.84	10.8	1.60	1.66
**Reading**	9.2	1.51	1.42	9.2	1.22	1.35
**Headaches**	0.0	1.83	1.51	0.0	1.66	1.33
**Concentration**	0.0	0.94	1.34	0.0	0.77	1.01
**Work**	0.0	1.23	1.33	0.0	0.97	1.14
**Driving**	44.6	1.08	1.32	44.6	0.89	1.09
**Sleeping**	0.0	1.58	1.71	0.0	1.63	1.67
**Recreation**	1.5	0.97	1.25	1.5	0.86	1.23

**Figure 2:**
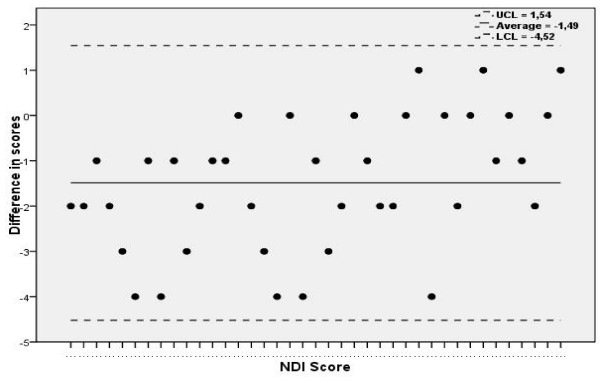
Bland and Altman graphic representation of the reliability of the NDI.

Internal consistency of the NDI exceeded the acceptable level resulting in a Cronbach's alpha: 0.85. Based on GROC, 46 patients were considered "stable" and were included in the test-retest analysis. The ICC value calculated from these patients was 0.93 (95% CI 0.84–0.97). The Bland and Altman analysis showed that the means of the difference were -1,49 ± 3,03 (Figure 2).

The exploratory factor analysis yielded 1 factor with Eigenvalue: 4.48 explaining 44.77% of variance. Loadings of all items are presented in Table 3.

**Table 3:** Factor loadings for the one-factor solution of the Greek version of the NDI

**Item**	**Factor**
**Pain intensity**	0.301
**Personal care**	0.514
**Lifting**	0.745
**Reading**	0.814
**Headaches**	0.371
**Concentration**	0.741
**Work**	0.720
**Driving**	0.780
**Sleeping**	0.629
**Recreation**	0.834

Regarding the analysis of responsiveness the Spearman correlation coefficient, as calculated for stable and improved patients, was 0.3 (P = 0.02). The calculations for sensitivity to change also revealed a SEM: 0.64 and an MDC: 1.78 (expressed in scale points). Based on the last value, 8.5% of patients had initial scores within 1 MDC distance from the best possible answer (no pain and no disability) revealing no ceiling effect according to the 15% criterion. Respectively, 0% of patients scored within 1 MDC distance from the worst possible answer (totally disabled) revealing no floor effect. Results from reliability and responsiveness analyses are summated in Table 4.

**Table 4:** Summary of reliability and responsiveness indices

***Property***	***Index***	***NDI***
**Reliability**	**Cronbach alpha**	0.85
	**Intraclass Correlation Coefficient**	0.93 (95%CI 0.84–0.97)
**Responsiveness**	**Spearman's correlation coefficient**	0.3 (P = 0.02)
	**Standard Error of Measurement**	0.64
	**Minimal Detectable Change**	1.78

## Discussion

The present study was conducted with the purpose of translating and validating the NDI in a Greek population with neck complaints. The translation procedure resulted in some modifications, with the purpose of increasing specificity of the Gr-NDI in detecting disabled patients due to neck pain. Swedish authors also described similar modifications [9]. Cultural equivalence was established using quality methods (interview with open-ended questions) resulting in a good content validity for the questionnaire.

The psychometric properties of the NDI were explored in a sample population with main characteristics: older age, low educational level, chronic neck pain (the majority of patients had previous episodes). The large amount of missing responses for 'driving' (44.6%) was not a translation issue since that finding is consistent with other studies [28]. Additionally, 6 patients (9.2%) who did not answer to the 'reading'item had previously stated to be illiterate, thus providing for the lack of translation problems. Finally, 7 patients (10.8%) who stated that they have difficulties in lifting due to their low back pain were considered as not answering this question. It is interesting that some patients mark an answer without mentioning the real cause of disability. Feedback with the GPs was determinative to avoid such biased answers. Perhaps, in instances of self-administration, written instructions and a space for the reasons to be given would be appropriate.

Removing items having low applicability in patients with certain demographical or clinical characteristics (driving in older ages, reading in illiterate, lifting and sleeping in co-morbidity), would result in loss of content validity of the questionnaire. However, low applicability raises the issue of dealing with missing data, increasingly discussed in the literature [29]. In order to avoid loss of power we applied a combination of two popular methods: case deletion and constant replacement [30].

High internal consistency of the Greek NDI (Cronbach alpha: 0.85) falls into the range of results from other studies (0.74–0.92). The very good test-retest reliability (ICC: 0.93) is comparable with the results of the Dutch study (ICC: 0.90) since they used similar methods to ours. Our findings are also consistent with the English, French, Swedish and Brazilian studies, although the methods used are varying. Factor analysis revealed one dimension, which is consistent with two other studies [31,10]. Nevertheless the percentage of variance explained in this factor solution is rather low (<50%) which could be considered as a limitation of our study.

Since the NDI is a condition-specific instrument, it is considered responsive to changes and thus appropriate for evaluative purposes. It is often used as an outcome measure in studies exploring the effectiveness of interventions, in patients with neck pain [32]. Checking the responsiveness of the Greek version of the NDI, we found significant correlation between Gr-NDI change scores and the GROC. This is in contrast with the study of Cleland [33], where they evaluated the responsiveness of the instrument in patients with cervical radiculopathy. Calculations of the MDC revealed that a change score of at least two points was required to demonstrate statistically important change. Since patients were rated as 'stable' or 'improved' using GROC, a difference of two points between scores could be assumed as having clinical importance. Based on the 15% criterion, the ability of the Gr-NDI to detect change over time was not constrained, thus making the interpretation of findings meaningful. Nevertheless, sensitivity to change of the questionnaire must also be explored in greater intervals since long-term outcomes are essential in estimating the effectiveness of interventions.

Another limitation of our study is that global ratings do not represent a standard way of assessing changes in functional status. Therefore definitions of clinically important changes could be inaccurate.

## Conclusion

We have accumulated enough evidence to show that the Greek version of the Neck Disability Index measures disability in activities of daily leaving in patients with neck pain in a reliable, valid and responsive manner. The questionnaire is considered a useful tool for research and clinical settings in Greek Primary Health Care. It is also appropriate for use in international studies since its psychometric properties are comparable with other versions validated in different countries.

## Abbreviations

NDI: Neck Disability Index; GPs: General Practitioners; GROC: Global Rating of Change; ICC: Intraclass Correlation Coefficient; SEM: Standard Error of Measurement; MDC: Minimal Detectable Change

## Competing interests

The authors declare that they have no competing interests.

## Authors' contributions

All authors read and approved the final manuscript. MNT participated in study design, forward translation, pretesting, carried out data entry, participated in statistical analysis and interpretation of data and wrote the final draft of the manuscript.

HTV provided appraisal and made suggestions during all stages of the translation process. He was also consulted during the validation process and revised the final draft of the manuscript. KNK participated in study design, forward translation and pretesting phases and has been involved in the revision of the final draft. MDA and ANP participated in the acquisition of data and the revision of the final draft. CDL conceived the study design, participated in the translation, pretesting and validation phases and revised the initial and the final draft of the manuscript.

## Pre-publication history

The pre-publication history for this paper can be accessed here:



## Supplementary Material

Additional file 1 Greek version of the Neck Disability Index. Click here for file
