# Supplementary material for: Translation of the Neck Disability Index and validation of the Greek version in a sample of neck pain patients
Source: BMC Musculoskelet Disord. 2008 Jul 22;9:106. doi: 10.1186/1471-2474-9-106 (PMC2492862; doi:10.1186/1471-2474-9-106)
Supplement: Additional file 1 — Greek version of the Neck Disability Index. [file 1471-2474-9-106-S1.pdf]

Δείκτης Ανικανότητας του Αυχένα

*Όνομα ασθενούς:*_______________________________ *# Αρχείο*_______ *Ημερομηνία:*__________

Παρακαλούμε διαβάστε τις οδηγίες:

Αυτό το ερωτηματολόγιο έχει σχεδιαστεί προκειμένου να δώσει στον ιατρό πληροφόρηση αναφορικά με τον τρόπο που ο πόνος στον αυχένα σας έχει επηρεάσει την ικανότητά σας να διαχειρίζεστε την καθημερινή σας ζωή. Παρακαλώ απαντήστε σε κάθε ερώτηση και σημαδέψτε σε κάθε μία μόνο ***ΕΝΑ κουτί*** *που σας ταιριάζει. Αντιλαμβανόμαστε ότι μπορεί να θεωρήσετε ότι δύο από τις απαντήσεις σε κάποια ερώτηση μπορεί να σας αφορούν, όμως παρακαλούμε* ***απλώς σημαδέψτε το κουτί που καλύτερα περιγράφει το πρόβλημά σας.***

ΜΕΡΟΣ 1- ΕΝΤΑΣΗ ΠΟΝΟΥ

☐Δεν έχω καθόλου πόνο στον αυχένα αυτή τη στιγμή.
☐Ο πόνος στον αυχένα είναι πολύ ήπιος αυτή τη στιγμή.
☐Ο πόνος στον αυχένα είναι μέτριος αυτή τη στιγμή.
☐Ο πόνος στον αυχένα είναι αρκετά σοβαρός αυτή τη στιγμή.
☐Ο πόνος στον αυχένα είναι πολύ σοβαρός αυτή τη στιγμή.
☐Ο πόνος στον αυχένα αυτή τη στιγμή είναι ότι χειρότερο έχω φανταστεί.

ΜΕΡΟΣ 2- ΠΡΟΣΩΠΙΚΗ ΦΡΟΝΤΙΔΑ (Πλύσιμο, ντύσιμο κ.λπ.)

☐Μπορώ να φροντίσω τον εαυτό μου φυσιολογικά χωρίς να προκαλώ περισσότερο πόνο στον αυχένα.
☐Μπορώ να φροντίσω τον εαυτό μου φυσιολογικά αλλά προκαλώ περισσότερο πόνο στον αυχένα.
☐Είναι επώδυνο να φροντίσω τον εαυτό μου και είμαι αργός/ή και προσεκτικός/ή.
☐Χρειάζομαι κάποια βοήθεια αλλά μπορώ να ανταποκριθώ στο μεγαλύτερο μέρος της προσωπικής μου φροντίδας.
☐Χρειάζομαι βοήθεια καθημερινά στα περισσότερα θέματα που αφορούν την προσωπική μου φροντίδα.
☐Δεν μπορώ να ντυθώ, πλένομαι με δυσκολία και παραμένω στο κρεβάτι.

ΜΕΡΟΣ 3- ΑΡΣΗ ΒΑΡΟΥΣ

☐Μπορώ να σηκώσω μεγάλα βάρη χωρίς περισσότερο πόνο στον αυχένα.
☐Μπορώ να σηκώσω μεγάλα βάρη αλλά αυτό προκαλεί περισσότερο πόνο στον αυχένα.
☐Ο πόνος στον αυχένα με εμποδίζει να σηκώσω μεγάλα βάρη από το πάτωμα, αλλά μπορώ να τα καταφέρω εάν είναι κατάλληλα τοποθετημένα, π.χ. πάνω σε ένα τραπέζι.
☐Ο πόνος στον αυχένα με εμποδίζει να σηκώσω μεγάλα βάρη από το πάτωμα, αλλά μπορώ να σηκώσω ελαφριά και μέτρια βάρη εάν είναι κατάλληλα τοποθετημένα.
☐Μπορώ να σηκώσω πολύ ελαφριά βάρη.
☐Δεν μπορώ να σηκώσω ή να μεταφέρω οτιδήποτε.

ΜΕΡΟΣ 4- ΔΙΑΒΑΣΜΑ

☐Μπορώ να διαβάσω όσο θέλω, χωρίς πόνο στον αυχένα μου.
☐Μπορώ να διαβάσω όσο θέλω, με λίγο πόνο στον αυχένα μου.
☐Μπορώ να διαβάσω όσο θέλω, με μέτριο πόνο στον αυχένα μου.
☐Δεν μπορώ να διαβάσω όσο θέλω, εξαιτίας μέτριου πόνου στον αυχένα μου.
☐Μετά βίας μπορώ να διαβάσω εξαιτίας δυνατού πόνου στον αυχένα μου.
☐Δεν μπορώ να διαβάσω καθόλου.

ΜΕΡΟΣ 5- ΠΟΝΟΚΕΦΑΛΟΙ

☐Δεν έχω καθόλου πονοκεφάλους.
☐Έχω ήπιους πονοκεφάλους που εμφανίζονται σπάνια.
☐Έχω μέτριους πονοκεφάλους που εμφανίζονται σπάνια.
☐Έχω μέτριους πονοκεφάλους που εμφανίζονται συχνά.
☐Έχω ισχυρούς πονοκεφάλους που εμφανίζονται συχνά.
☐Έχω πονοκεφάλους σχεδόν πάντα.

ΜΕΡΟΣ 6- ΣΥΓΚΕΝΤΡΩΣΗ

☐Μπορώ να συγκεντρωθώ πλήρως όταν το θελήσω, χωρίς καμία δυσκολία.
☐Μπορώ να συγκεντρωθώ πλήρως όταν το θελήσω, με μικρή δυσκολία.
☐Έχω μέτριο βαθμό δυσκολίας στο να συγκεντρωθώ όταν το θελήσω.
☐Έχω αρκετή δυσκολία στο να συγκεντρωθώ όταν το θελήσω.
☐Έχω πολύ μεγάλη δυσκολία στο να συγκεντρωθώ όταν το θελήσω.
☐Δεν μπορώ να συγκεντρωθώ καθόλου.

ΜΕΡΟΣ 7- ΕΡΓΑΣΙΑ

☐Μπορώ να εργαστώ όσο θέλω.
☐Μπορώ να κάνω τη συνηθισμένη εργασία μου, αλλά όχι κάτι περισσότερο.
☐Μπορώ να κάνω το μεγαλύτερο μέρος από τη συνηθισμένη εργασία μου, αλλά όχι κάτι περισσότερο.
☐Δεν μπορώ να κάνω τη συνηθισμένη εργασία μου.
☐Μετά βίας μπορώ να εργαστώ.
☐Δεν μπορώ να εργαστώ καθόλου.

ΜΕΡΟΣ 8- ΟΔΗΓΗΣΗ

☐Μπορώ να οδηγήσω το αυτοκίνητό μου χωρίς καθόλου πόνο στον αυχένα.
☐Μπορώ να οδηγήσω το αυτοκίνητό μου όσο θελήσω, με ελαφρύ πόνο στον αυχένα μου.
☐Μπορώ να οδηγήσω το αυτοκίνητό μου όσο θελήσω, με μέτριο πόνο στον αυχένα μου.
☐Δεν μπορώ να οδηγήσω το αυτοκίνητό μου όσο θελήσω, εξαιτίας μέτριου πόνου στον αυχένα μου.
☐Μετά βίας μπορώ να οδηγήσω, εξαιτίας δυνατού πόνου στον αυχένα μου.
☐Δεν μπορώ να οδηγήσω το αυτοκίνητό μου καθόλου.

ΜΕΡΟΣ 9- ΥΠΝΟΣ

☐Δεν έχω πρόβλημα με τον ύπνο.
☐Ο ύπνος μου είναι ελαφρά διαταραγμένος (λιγότερο από 1 ώρα άυπνος/η).
☐Ο ύπνος μου είναι ήπια διαταραγμένος (1-2 ώρες άυπνος/η).
☐Ο ύπνος μου είναι μέτρια διαταραγμένος (2-3 ώρες άυπνος/η).
☐Ο ύπνος μου είναι πολύ διαταραγμένος (3-5 ώρες άυπνος/η).
☐Ο ύπνος μου είναι εντελώς διαταραγμένος (5-7 ώρες άυπνος/η).

ΜΕΡΟΣ 10- ΨΥΧΑΓΩΓΙΑ

☐Μπορώ να ασχοληθώ με όλες τις ψυχαγωγικές μου δραστηριότητες, χωρίς καθόλου πόνο στον αυχένα.
☐Μπορώ να ασχοληθώ με όλες τις ψυχαγωγικές μου δραστηριότητες, με κάποιο πόνο στον αυχένα.
☐Μπορώ να ασχοληθώ με τις περισσότερες αλλά όχι με όλες τις ψυχαγωγικές μου δραστηριότητες, εξαιτίας πόνου στον αυχένα μου.
☐Μπορώ να ασχοληθώ με λίγες από τις συνήθεις ψυχαγωγικές μου δραστηριότητες, εξαιτίας πόνου στον αυχένα μου.
☐Μετά βίας συμμετέχω σε ψυχαγωγικές δραστηριότητες, εξαιτίας πόνου στον αυχένα μου.
☐Δεν μπορώ καθόλου να συμμετέχω σε ψυχαγωγικές δραστηριότητες.

H. Vernon D.C. & S. Mior D.C. © 1991

Για τη μετάφραση, προσαρμογή και στάθμιση του ερωτηματολογίου στην ελληνική γλώσσα έχει δοθεί άδεια στην *Κλινική Κοινωνικής και Οικογενειακής Ιατρικής του τμήματος Ιατρικής, Πανεπιστημίου Κρήτης*. Υπεύθυνοι είναι οι: *Χρήστος Λιονής, Αναπλ. Καθ. Κοιν. & Οικ. Ιατρ. Παν. Κρήτης. και Μαριάννα Τρούλη, Φυσ/τρια, Μετ. Φοιτ. στη Δ.Υ. & Δ.Υ.Υ.*
